# *Brevibacillus laterosporus* BL1, a promising probiotic, prevents obesity and modulates gut microbiota in mice fed a high-fat diet

**DOI:** 10.3389/fnut.2022.1050025

**Published:** 2022-11-24

**Authors:** Guangying Weng, Jian Huang, Xianyong Ma, Min Song, Yulong Yin, Dun Deng, Jinping Deng

**Affiliations:** ^1^Guangdong Provincial Key Laboratory of Animal Nutrition Regulation, South China Agricultural University, Guangzhou, Guangdong, China; ^2^State Key Laboratory of Livestock and Poultry Breeding, Key Laboratory of Animal Nutrition and Feed Science in South China, Ministry of Agriculture and Rural Affairs, Guangdong Provincial Key Laboratory of Animal Breeding and Nutrition, Institute of Animal Science, Guangdong Academy of Agricultural Sciences, Guangdong Engineering Technology Research Center of Animal Meat Quality and Safety Control and Evaluation, Guangzhou, China; ^3^Institute for Quality & Safety and Standards of Agricultural Products Research, Jiangxi Academy of Agricultural Sciences, Nanchang, Jiangxi, China

**Keywords:** *Brevibacillus laterosporus*, obesity, gut microbiota, lipid metabolism, high-fat diet

## Abstract

**Scope:**

Probiotics are a potential preventive strategy for obesity. However, with discrete efficacy and limited species of probiotics, there is a demand for novel strains with excellent anti-obesity properties. This study aimed to investigate the effects of Brevibacillus laterosporus BL1 on preventing obesity in high-fat diet (HFD)-fed mice.

**Methods and results:**

C57BL/6 male mice were randomly assigned to four groups (*n* = 10) and fed a control diet, HFD, HFD plus *B. laterosporus* BL1, and HFD plus supernatant of *B. laterosporus* BL1, respectively for 8 weeks. The results showed that prophylactic *B. laterosporus* BL1 treatment reduced body weight gain by 41.26% in comparison to the HFD group, and this difference was accompanied by a reduction in body fat mass and the weight of inguinal white adipose tissues and epididymal white adipose tissue (−33.39%, −39.07%, and −43.75%, respectively). Moreover, the *B. laterosporus* BL1-mediated improvements in lipid profile, insulin resistance, and chronic inflammation were associated with the regulation of gene expression related to lipid metabolism and enhancement of brown adipose tissue thermogenesis. Particularly, *B. laterosporus* BL1 intervention significantly improved HFD-induced gut flora dysbiosis, as evidenced by a reverse in the relative abundance of Bacillota and Bacteroidota, as well as an increase in the relative abundance of bacteria that produce short-chain fatty acids (SCFAs), which in turn increased SCFAs levels.

**Conclusion:**

Our findings found for the first time that *B. laterosporus* BL1 may be a promising probiotic for prevention of obesity associated with the regulation of gut microbiota.

## Graphical Abstract

*Brevibacillus laterosporus* BL1 treatment effectively reduced fat accumulation, proinflammatory cytokines, and insulin resistance, alleviated BAT whitening, and improved glucose tolerance and lipid profiles in HFD-induced obese mice. *B. laterosporus* BL1 intervention also modulated the HFD-induced structural and componential alteration of gut microbiota, as evidenced by a decrease in the relative abundance of Bacillota and *Faecalibaculum* and an increase in the relative abundance of Bacteroidota and norank_f_*Muribaculaceae*, and this alteration was accompanied by an elevation on short-chain fatty acids (SCFAs) production. The alleviation of obesity parameters with *B. laterosporus* BL1 treatment was associated with the modulation of gut microbiota, especially the increase in SCFA levels. 
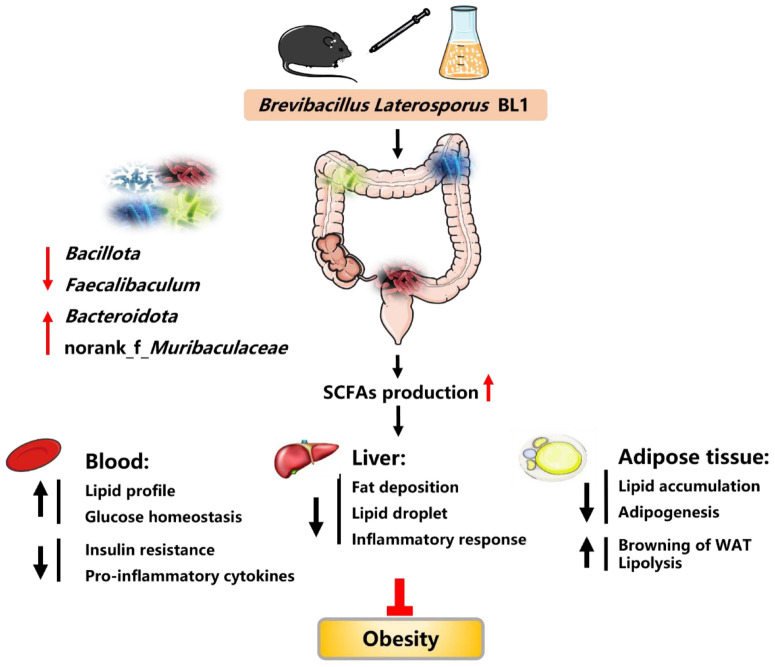

## Introduction

The incidence of obesity has risen alarmingly in recent years, and it is starting to overtake malnutrition and infectious disease as the major threat to global public health (1, 2). Obesity is characterized by an excessive or abnormal ectopic lipid accumulation and results from a serious imbalance between energy intake and energy consumption (3). There is a wealth of data demonstrating that obesity is responsible for the increased risk of numerous chronic diseases, such as cardiovascular disease, atherosclerosis, and diabetes (4, 5). Several factors such as genes, environment, dietary patterns, and lifestyle have been identified to regulate obesity and its related metabolic diseases (6). In this context, several strategies and therapies have been applied to prevent and treat obesity, including pharmacotherapy, surgery, and lifestyle modification. However, with high risk and recurrence rates, it is difficult to achieve long-term goals in clinical applications using traditional therapies (7, 8). Therefore, much research has been devoted to developing effective bioactive substances from natural biological sources for obesity prevention and control.

Probiotics are active microorganisms that help the host’s health after adequate administration (9). As the most widely available probiotics, *Lactobacillus* and *Bifidobacteria* have been proven to exhibit favorable effects on lipid metabolic disorders including obesity and NAFLD through regulation of energy metabolism, inflammatory response, and intestinal barrier function (10–12). For example, administration of *Limosilactobacillus* (*Lactobacillus*) *fermentum* CECT5716 (5 × 10^8^ CFU/d, 11 weeks) resulted in an obvious anti-obesity effect *via* amelioration of endothelial and intestinal barrier dysfunction in HFD-induced obese mice (13). Likewise, intervention with *Bifidobacteria breve* B-3 (10^8^ or 10^9^ CFU/d, 8 weeks) in HFD-feeding mice dose-dependently suppressed body weight gain and fat accumulation (14). Despite these positive outcomes, a recent systematic study showed that the species of probiotics that are effective in the prevention or treatment of adiposis are scarce and limited probiotics produce a discrete benefit in clinical applications (15). For this reason, there is an urgent demand to identify novel strains with anti-obesity properties and the functional mechanisms involved in their actions.

Over time, evidence has accumulated in support of the important role that gut microbiota plays in host energy metabolism and the development of obesity (3, 16, 17). A transformation from a low-fat diet to an HFD changed gut microbial communities within 24–48 h in mice and humans, leading to obesity and its related metabolic disorders (18–20). Probiotics have been shown to improve gut microbiota composition and diversity, as manifested by enriching specific health-promoting bacteria *via* competitive exclusion of pathogens and producing antimicrobial substances, thus improving gut dysbiosis and host metabolic homeostasis (21, 22). In addition, probiotics may elevate short-chain fatty acids (SCFAs) production, which functions not only as energy sources to enhance the integrity of the intestinal epithelial barrier but as signaling molecules to enter the systemic circulation and directly regulate lipid dysmetabolism (23, 24). Therefore, the modulation of gut microbiota with probiotics should get more specific attention, as these may represent a preventive or therapeutic strategy for obesity and its related metabolic syndromes.

*Brevibacillus laterosporus*, a pathogen of invertebrates, produces various antimicrobial substances such as polyketides, non-ribosomal peptides, antibiotics, and chitinase, resulting in broad-spectrum antimicrobial activity (25, 26). In 2013, the Ministry of Agriculture and Rural Affairs of the People’s Republic of China published a notification to include *B. laterosporus* in the catalog of feed additives. However, to our knowledge, few studies have explored the beneficial effects of *B. laterosporus* in livestock and poultry (27). Our pre-experiment performed in a porcine model implied that *B. laterosporus* has the potential to improve abnormal fat deposition, but the underlying mechanism remained unclear. Moreover, considering its excellent antimicrobial activity, we hypothesized that *B. laterosporus* could regulate gut microbiota dysbiosis and ameliorate excessive fat deposition. To confirm this hypothesis, we isolated a novel *B. laterosporus* strain BL1 from a healthy earthworm intestine and investigated the effects of *B. laterosporus* BL1 on obesity in HFD-fed mice for the first time, with particular emphasis on the potential role of intestinal flora modulation as a novel preventive approach. We also evaluated its effects on fat deposition, serum lipids, insulin resistance, chronic inflammation, intestinal flora composition, and colonic SCFAs in HFD-fed mice and further explored the correlation between key gut microbial taxa and SCFAs/obesity-related parameters.

## Materials and methods

### Bacterial strain and culture

*Brevibacillus laterosporus* BL1 strain was originally isolated from a healthy earthworm intestine and stored in Guangdong Microbial Culture Collection Center, China (GDMCC 62699). The neighbor-joining phylogenetic tree of *B. laterosporus* BL1 was added in Supplementary Figure 1. The strain was incubated aerobically in Luria–Bertani media for 24 h at 37°C while being vigorously shaken at 180 r/min. For administration to mice, pure bacterial cells were collected after being centrifuged (6,000 *g*, 10 min, 4°C) and cleansed twice with sterile saline, and adjusted to 5 × 10^9^ CFU/mL. When the bacterial concentration reached 5 × 10^9^ CFU/mL, the culture supernatant was centrifuged (6,000 *g*, 10 min, 4°C) and filtered through 0.22 μm filters to prepare cell-free supernatant.

### Animals and diets

The South China Agricultural University’s Animal Care and Use Committee approved the use of animals in the experiments, which were carried out following the acknowledged guidelines for animal care (Authorization Number: 2021C097).

Forty 5-week-old male C57BL/6 mice were purchased from (Zhuhai BesTest Bio-Tech Co., Ltd., Zhuhai, China) and individually kept in a controlled environment (24 ± 2°C, 45–60% humidity and 12/12 h light/dark cycle) with free access to drinking water and food throughout the experiment. Corncob granules obtained from (Slac Laboratory Animal Co., Ltd., Shanghai, China) were used as bedding for mice. Both the control diet (XTCON50J) and the HFD (XTHF60) were purchased from (Xietong Organism Co., Led., Nanjing, China) and the composition and nutrient levels of diets are listed in Supplementary Table 1. After 1 week of adaptation, the mice were divided randomly into four groups (*n* = 10 per group) as follows: CON group (control diet, 10% kcal from fat), HFD group (high-fat diet, 60% kcal from fat), BL group (HFD along with *B. laterosporus* BL1, 1 × 10^9^ CFU/day per mouse), and BLs group (HFD along with supernatant of *B. laterosporus* BL1, 200 μL/day per mouse). The mice in CON and HFD groups were treated with 200 μL sterile saline *via* oral gavage per day at 8:00 a.m. Body weight (BW) and food intake were recorded weekly. At the end of the 8-week treatment, body fat mass was measured using a QMR body composition analyzer (Shanghai Electronic Technology CO., LTD., Shanghai, China). The infra-red thermal images and temperature of brown adipose tissue (BAT) were measured by an infra-red detector (FLIR Systems, Inc.). Blood samples were obtained through orbital blooding. Liver, inguinal white adipose tissues (iWAT), and epididymal white adipose tissue (eWAT) were weighed. Subsequently, liver, iWAT, BAT, and colonic digesta samples were collected for further analysis (28, 29).

### Intraperitoneal glucose and insulin tolerance tests

At week 7, intraperitoneal glucose tolerance tests (IGTT) and insulin tolerance tests (ITT) were performed. A glucometer (Sinocare Biological Transmission Co., Ltd., Changsha, China) was used to detect the blood glucose levels in the tail vein blood. For IGTT analysis, mice fasted overnight were intraperitoneally injected with glucose of 2 g/kg BW, followed by measuring blood glucose levels at 0, 15, 30, 60, 90, and 120 min. For ITT analysis, blood glucose concentrations were measured at 0, 15, 30, 60, 90, and 120 min after being injected intraperitoneally with insulin of 0.75 U/kg BW to mice fasted (6 h). The trapezoidal rule was used to compute the integrated glucose areas under the curve (AUC). The following equation was used to calculate the homeostasis model of insulin resistance index (HOMA-IR): HOMA-IR = basal glucose × basal insulin/22.5 (21).

### Biochemical analysis

The blood samples were centrifuged (3,000 rpm, 4°C, 10 min) to separate the serum. Serum glucose, triglyceride (TG), total cholesterol (T-CHO), high-density lipoprotein cholesterol (HDL-C), and low-density lipoprotein cholesterol (LDL-C) were detected using commercial kits (Nanjing Jiancheng Bioengineering Institute, Nanjing, China). Serum concentrations of insulin, interleukin-β (IL-1β), and tumor necrosis factor-α (TNF-α) were measured using commercial ELISA kits from (Meimian Biotechnology Company, Jiangsu, China).

### Histological analysis

The fresh iWAT, eWAT, and BAT samples were fixed with 4% paraformaldehyde, embedded in paraffin, sliced into 5–7 μm, and stained with Hematoxylin and eosin (H and E) following standard procedures (29). The ImageJ software (NIH, USA) was used to calculate the adipocyte size. The oil red O staining of liver tissues was carried out by previously described methods (28).

### Quantitative PCR analysis

Using a TRizol reagent (Takara Biotechnology, Dalian, China) to extract total RNA from selected tissues and a Synthesis Kit (Takara Biotechnology) to produce cDNA according to the manufacturer’s protocols. Following the manufacturer’s instructions, real-time RT-PCR was performed in duplicate using a CFX96 Real-time PCR Detection System (Bio-Rad Laboratories, Hercules, CA, USA). The relative expression of target genes was calculated using the 2^–△△Ct^ method, which normalized to the housekeeping gene β-actin (30–32). The target genes, including sterol regulatory element binding protein 1 (SREBP1), peroxisome proliferators-activated receptor γ (PPARγ), fatty acid synthase (FAS), cluster of differentiation 36 (CD36), hormone-sensitive lipase (HSL), carnitine palmitoyltransferase-1 (CPT-1), uncoupling protein 1 (UCP1), peroxisome proliferator-activated receptor γ coactivator 1α (PGC-1α), CCAAT/enhancer binding protein α (C/EBPα), PR domain-containing 16 (PRDM16), IL-1β, TNF-α, interleukin 6 (IL-6), and interferon-γ (IFN-γ) and their primer sequences are shown in Supplementary Table 2.

### Gut microbiota analysis

The microbial genomic DNA of colonic digesta samples was extracted using the QIAamp-DNA Stool Mini Kit (Qiagen, Hilden, Germany). The amplification of DNA samples used common primers of variable region V3-V4 of the bacterial 16S rRNA gene with primers 338F and 806R (forward primer, 5′-ACTCCTACGGGAGGCAGCAG-3′; reverse primer, 5′-GGACTACHVGGGTWTCTAAT-3′). All the samples were sequenced using the Illumina MiSeq PE300 platform at Majorbio Biotechnology Co., Ltd. (Shanghai, China). After sequencing, raw sequence reads were quality filtered with fastp (v0.19.6) (33) and merged with FLASH (v1.2.11) (34). Then the DADA2 plugin in the Qiime2 pipeline was used to de-noise the high-quality sequences into amplicon sequence variants (ASVs) according to the recommended parameters. The taxonomy of ASVs was analyzed by the Naive Bayes Classifier implemented in QIIME2 based on the silva138/16s_bacteria database. Subsequently, the diversity and richness of microbial communities were evaluated by alpha diversity indices (Sobs, Shannon, and Simpson) using Mothur (v1.30.2). The similarity among the microbial community of different samples was analyzed by Principal coordinate analysis (PCoA) based on Bray–Curtis distance using the Vegan (v2.5-3) package. Spearman correlation analysis was carried out to explore the correlations between key intestinal microbiota and metabolites/obesity-related parameters.

### Short-chain fatty acids analysis

The concentrations of SCFAs were determined using the GCMS-QP2020 system (Shimadzu, Tokyo, Japan) as previously described (35). Briefly, the colonic digesta samples were homogenized in ultra-pure water, sonicated, then centrifuged to obtain supernatant. Subsequently, metaphosphoric acid solution, anhydrous sodium sulfate, and methyl tert-butyl ether were added to acidification, salting out, and extraction, respectively. After centrifuging and filtering, the organic phase was collected for GC-MS analysis. The gas chromatography equipped with a DB-FFAP capillary column and a flame ionization detector was utilized for chromatographic separation (injection port temperature: 250°C, carrier gas: helium, total run time: 18 min). The temperature programing is as follows: The initial temperature was 80°C for 2 min and raised to 150°C at 10°C/min for 2 min, and to 180°C at 15°C/min for 5 min. The quantification of SCFAs was performed by an external standard method as previously described (36).

### Statistical analysis

The GraphPad Prism 8.0 software (GraphPad Software Inc., San Diego, CA, USA) was utilized for statistical analyses. One-way analysis of variance (ANOVA) with Tukey’s test was used to analyze all experimental data. All data were expressed as the means ± SEM. Differences were considered statistically significant at *p*-values<0.05 and tendencies were designated as having *p*-values<0.10.

## Results

### *Brevibacillus laterosporus* BL1 alleviates body weight and lipid accumulation in high-fat diet-fed mice

**FIGURE 1:**
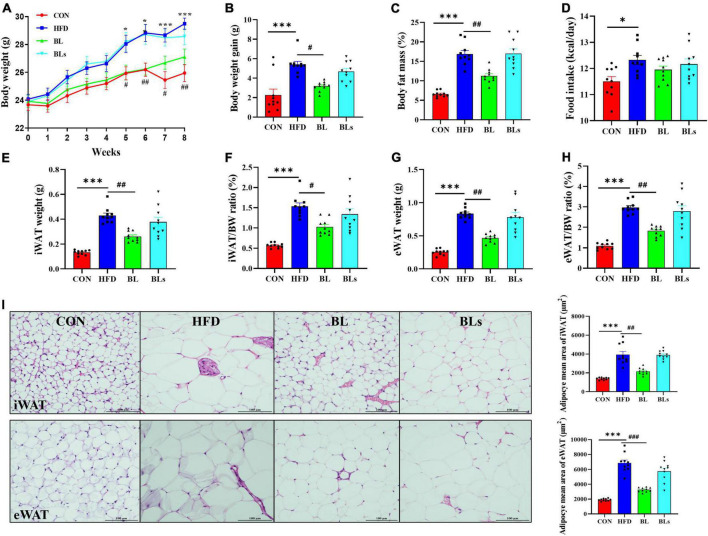
*Brevibacillus laterosporus* BL1 treatment reduced body weight gain and fat accumulation in HFD-fed mice. **(A)** Body weight, **(B)** body weight gain, **(C)** body fat mass, **(D)** food intake, **(E)** iWAT weight, **(F)** iWAT/BW ratio, **(G)** eWAT weight, **(H)** eWAT/BW ratio, **(I)** H and E staining of iWAT and eWAT sections. Scale bar = 100 μm. Data are presented as mean ± SEM (*n* = 10) and analyzed using one-way ANOVA. Significant differences between HFD and CON are indicated by *p < 0.05 and ***p < 0.001. Significant differences between HFD and BL are indicated by ^#^*p* < 0.05, ^##^*p* < 0.01, and ^###^*p* < 0.001.

As shown in Figure 1, relative to the CON group, HFD-feeding significantly increased body weight (control diet: 25 ± 1.62; HFD: 29.49 ± 1.08; *p* < 0.001). As expected, *B. laterosporus* BL1 administration decreased body weight gain by 41.26% in obese mice (*p* < 0.05), leading to significantly lower body weight in the 8th week (*p* < 0.01). However, the supernatant of *B*. *laterosporus* BL1 coadministration failed to reduce body weight gain in mice fed with HFD (Figures 1A,B).

Similarly, HFD-fed mice significantly increased body fat mass compared with CON-fed mice (*p* < 0.001), while the body fat mass was reduced by 33.39% in the BL group (*p* < 0.01) (Figure 1C). Interestingly, there was no obvious difference in food intake between the HFD and BL groups, indicating that the anti-obesity effect of *B*. *laterosporus* BL1 treatment was not attributable to reduced food consumption (Figure 1D). As presented in Figures 1E–H, the iWAT and eWAT weights as well as their ratios to body weight were markedly elevated by the HFD but were significantly reduced by *B*. *laterosporus* BL1 treatment (−39.07%, *p* = 0.0017; −43.75%, *p* = 0.0013; −33.33%, *p* = 0.0113; −38.50%, *p* = 0.0015, respectively). Moreover, HFD significantly increased the mean adipocyte sizes of iWAT and eWAT in comparison with control mice, whereas *B*. *laterosporus* BL1 treatment markedly reduced these values (*p* < 0.01) (Figure 1I).

In addition, although there was no difference in liver weight among any group, oil red O staining results showed marked lipid accumulation in HFD-fed mice, whereas this accumulation was attenuated by *B*. *laterosporus* BL1 supplementation (Supplementary Figure 2). Taken together, these results suggested that *B*. *laterosporus* BL1 treatment may alleviate the obesity phenotypes induced by HFD in mice, however, these effects were not observed with the intervention of supernatant of *B*. *laterosporus* BL1.

### *Brevibacillus laterosporus* BL1 improves dyslipidemia and pathoglycemia in high-fat diet-fed mice

**FIGURE 2:**
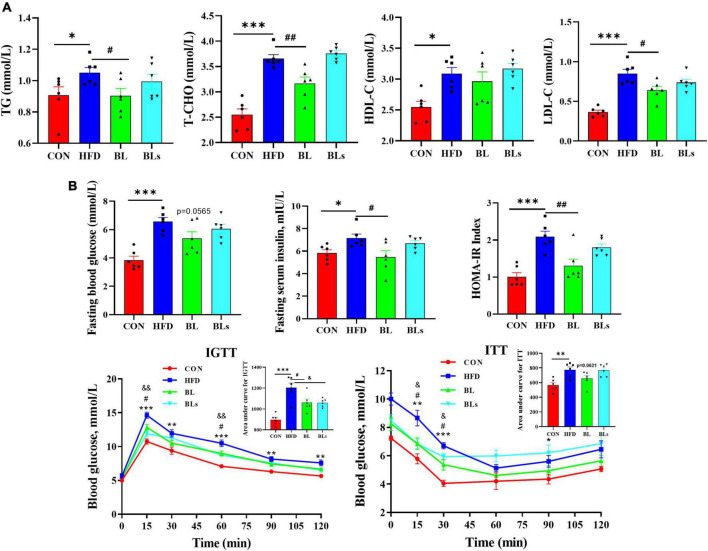
*Brevibacillus laterosporus* BL1 treatment improved serum lipid profile and insulin resistance in HFD-fed mice. **(A)** Serum concentrations of TG, T-CHO, HDL-C, and LDL-C, **(B)** serum concentrations of fasting blood glucose and insulin, IGTT and ITT, curves of blood glucose levels, and the calculated area under curve values. Data are presented as mean ± SEM (*n* = 6) and analyzed using one-way ANOVA. Significant differences between HFD and CON are indicated by *p < 0.05 and ***p < 0.001. Significant differences between HFD and BL are indicated by ^#^*p* < 0.05 and ^##^*p* < 0.01. Significant differences between HFD and BLs are indicated by ^&^*p* < 0.05.

To evaluate the potential of *B*. *laterosporus* BL1 to regulate hyperlipidemia, lipid concentrations in serum were measured (Figure 2A). Relative to the control group, serum levels of TG, T-CHO, HDL-C, and LDL-C were significantly higher in HFD-fed mice (*p* < 0.05), demonstrating that the HFD group had lipid dysmetabolism. *B*. *laterosporus* BL1 treatment strikingly reduced serum concentrations of TG, T-CHO, and LDL-C (*p* < 0.05) but had no effect on serum HDL-C level. However, there were no alleviative effects on hyperlipidemia induced by HFD in the BLs group.

As obesity is closely correlated with hyperglycemia and hyperinsulinemia, the concentrations of fasting blood glucose and fasting serum insulin were detected. As presented in Figure 2B, HFD treatment showed dramatic increases in glucose and insulin levels and HOMA-IR values relative to control mice, while these decreased by *B*. *laterosporus* BL1 intervention. In addition, the mice in the BL group demonstrated lower area under curve values in the IGTT and ITT relative to those in the HFD group, which further suggests that *B*. *laterosporus* BL1 ameliorated systemic glucose tolerance and insulin resistance induced by HFD. Collectively, these data suggested a protective effect of *B. laterosporus* BL1 against HFD-induced dyslipidemia and pathoglycemia.

### *Brevibacillus laterosporus* BL1 attenuates secretion of proinflammatory cytokines in high-fat diet-fed mice

**FIGURE 3:**
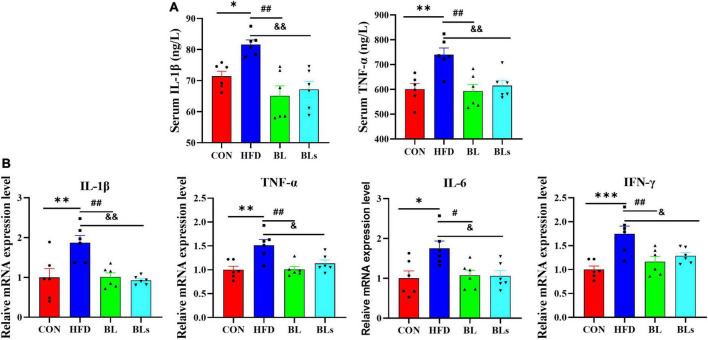
*Brevibacillus laterosporus* BL1 treatment alleviated the secretion of proinflammatory cytokines in HFD-fed mice. **(A)** Serum levels of IL-1β and TNF-α, **(B)** relative mRNA expression levels of IL-1β, TNF-α, IL-6, and IFN-γ were determined using real-time PCR in the liver. Data are presented as mean ± SEM (*n* = 6) and analyzed using one-way ANOVA. Significant differences between HFD and CON are indicated by *p < 0.05, **p < 0.01, and ***p < 0.001. Significant differences between HFD and BL are indicated by ^#^*p* < 0.05 and ^##^*p* < 0.01. Significant differences between HFD and BLs are indicated by ^&^*p* < 0.05 and ^&&^*p* < 0.01.

Low-grade chronic inflammation is strongly correlated with obesity, which has been shown to induce obesity-related glucose tolerance and insulin resistance (37). To examine whether *B. laterosporus* BL1 administration ameliorated chronic inflammation in HFD-fed mice, we further analyzed proinflammatory cytokines concentrations in serum as well as hepatic mRNA expression of proinflammatory cytokines. Serum IL-1β and TNF-α levels (*p* < 0.05) markedly elevated in HFD-fed mice relative to the control group (Figure 3A), while both bacteria and supernatant of *B. laterosporus* BL1 treatment markedly decreased IL-1β and TNF-α levels (*p* < 0.01). Moreover, as presented in Figure 3B, HFD significantly upregulated hepatic mRNA expression of IL-1β, TNF-α, IL-6, and IFN-γ, whereas the expressions of these proinflammatory cytokines were significantly downregulated in the BL and BLs groups (*p* < 0.05). These data together indicated that supplementation with either the bacteria or supernatant of *B. laterosporus* BL1 may have anti-inflammatory effects in HFD-fed mice.

### *Brevibacillus laterosporus* BL1 regulates the expression of lipid metabolism related genes in high-fat diet-fed mice

**FIGURE 4:**
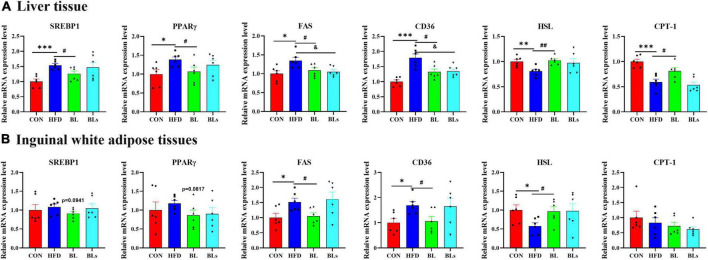
*Brevibacillus laterosporus* BL1 treatment regulated the mRNA expression levels of lipid metabolism-related genes in HFD-fed mice. **(A,B)** Relative mRNA expression levels of SREBP1, PPARγ, FAS, CD36, HSL, and CPT-1 in the liver and inguinal white adipose tissue. Data are presented as mean ± SEM (*n* = 6) and analyzed using one-way ANOVA. Significant differences between HFD and CON are indicated by *p < 0.05, **p < 0.01, and ***p < 0.001. Significant differences between HFD and BL are indicated by ^#^*p* < 0.05 and ^##^*p* < 0.01. Significant differences between HFD and BLs are indicated by ^&^*p* < 0.05.

In the liver, the HFD significantly upregulated mRNA expression of lipogenic genes (SREBP1, PPARγ, and FAS) and a lipid uptake gene (CD36) and downregulated mRNA expression of lipolytic genes (HSL and CPT-1) in comparison with the control diet (*p* < 0.05). However, the upregulation of SREBP1, PPARγ, FAS, and CD36 and the downregulation of HSL and CPT-1 induced by HFD were significantly reversed by *B. laterosporus* BL1 treatment (*p* < 0.05). Also, the supernatant of *B. laterosporus* BL1 administration downregulated FAS and CD36 mRNA expression in HFD-fed mice (*p* < 0.05) (Figure 4A).

In the iWAT, mice fed with HFD significantly upregulated FAS and CD36 mRNA expression and significantly downregulated HSL mRNA expression relative to mice fed the control diet (*p* < 0.05). Mice treated with *B. laterosporus* BL1, but not supernatant of *B. laterosporus* BL1, exhibited downregulation of SREBP1, PPARγ, FAS, and CD36 mRNA expression, and upregulation of HSL mRNA expression. CPT-1 mRNA expression did not differ significantly among the four groups (Figure 4B). Overall, these data demonstrated that *B. laterosporus* BL1 exerted a protective effect against lipid accumulation through decreasing lipogenesis and increasing lipolysis in HFD-induced obese mice, and this effect was not exerted by the supernatant of *B. laterosporus* BL1.

### *Brevibacillus laterosporus* BL1 triggers brown adipose tissue browning in high-fat diet-fed mice

**FIGURE 5:**
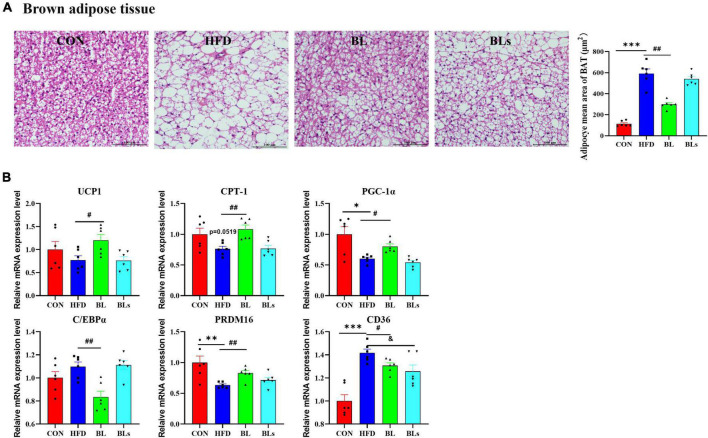
*Brevibacillus laterosporus* BL1 treatment triggered BAT browning in HFD-fed mice. **(A)** H and E staining of brown adipose tissue sections, scale bar = 100 μm, **(B)** relative mRNA expression levels of UCP-1, CPT-1, PGC-1α, C/EBPα, PRDM16, and CD36 in brown adipose tissue. Data are presented as mean ± SEM (*n* = 6) and analyzed using one-way ANOVA. Significant differences between HFD and CON are indicated by *p < 0.05, **p < 0.01, and ***p < 0.001. Significant differences between HFD and BL are indicated by ^#^*p* < 0.05 and ^##^*p* < 0.01. Significant differences between HFD and BLs are indicated by ^&^*p* < 0.05.

The adipocyte size of BAT was the largest in the HFD group (Figure 5A), leading to lipid accumulation (that is, whitening), whereas *B. laterosporus* BL1 treatment prevented HFD-induced whitening (*p* < 0.05). Moreover, *B. laterosporus* BL1 treatment significantly increased the thermogenesis and temperature of BAT (Supplementary Figure 3). Consistently, relative to the HFD group, *B. laterosporus* BL1 administration significantly increased the mRNA expression levels of UCP1 (thermogenesis), CPT-1, PGC-1α (mitochondrial biogenesis), and PRDM16 (adipocyte browning), and significantly reduced the mRNA expression levels of C/EBPα (lipogenesis) and CD36 in BAT (*p* < 0.05) (Figure 5B). These results suggested that *B. laterosporus* BL1 triggered BAT browning to suppress lipid accumulation in mice with HFD-induced obesity.

### *Brevibacillus laterosporus* BL1 modulates gut microbiota composition in high-fat diet-fed mice

**FIGURE 6:**
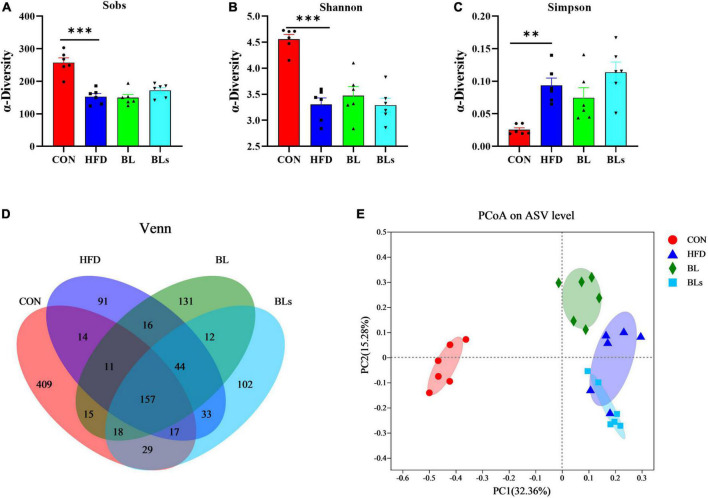
*Brevibacillus laterosporus* BL1 treatment altered gut microbiota diversity and composition in HFD-fed mice. **(A–C)** Sobs, Shannon, and Simpson indices in α-diversity analysis, **(D)** Venn diagrams showing the observed overlap of ASVs, **(E)** PCoA plot analysis from each sample. Data are presented as mean ± SEM (*n* = 6) and analyzed using one-way ANOVA. Significant differences between HFD and CON are indicated by **p < 0.01 and ***p < 0.001.

To determine the effects of *B. laterosporus* BL1 on the intestinal microbiota compositions of HFD-fed mice, the bacterial 16S rRNA V3–V4 region was sequenced. In the current study, an average of 60,433 clean reads were obtained from each sample (*n* = 6), and a total of 1,099 ASVs were obtained with the sequence denoising approach (Supplementary Table 3). The rarefaction curves for all samples exhibited clear asymptotes, indicating a near-complete sampling of the community (Supplementary Figure 4). As expected, mice fed with HFD showed lower richness and diversity of microbial community, as evidenced by lower Sobs, Shannon, and Simpson indices relative to the CON group. However, there was no obvious difference in α-diversity between the HFD and BL groups, indicating that *B. laterosporus* BL1 treatment did not significantly alter the taxa richness (Figures 6A–C). Venn diagrams (Figure 6D) show the shared and unique ASVs in all the treatment groups. A total of 157 of 1,099 ASVs overlapped among the four groups, with the CON, HFD, BL, and BLs groups having 409, 91, 131, and 102 specific ASVs respectively, which was consistent with the microbial community diversity. To better explore overall differences in intestinal microbiota structure and composition in all groups, the Bray–Curtis distance-based PCoA analysis was performed (Figure 6E, and PC1 and PC2 were 32.36–15.28%, respectively). The results revealed that mice in the CON, HFD, and BL groups showed distinct clustering of bacteria composition, while the BLs groups clustered closely with the HFD groups.

**FIGURE 7:**
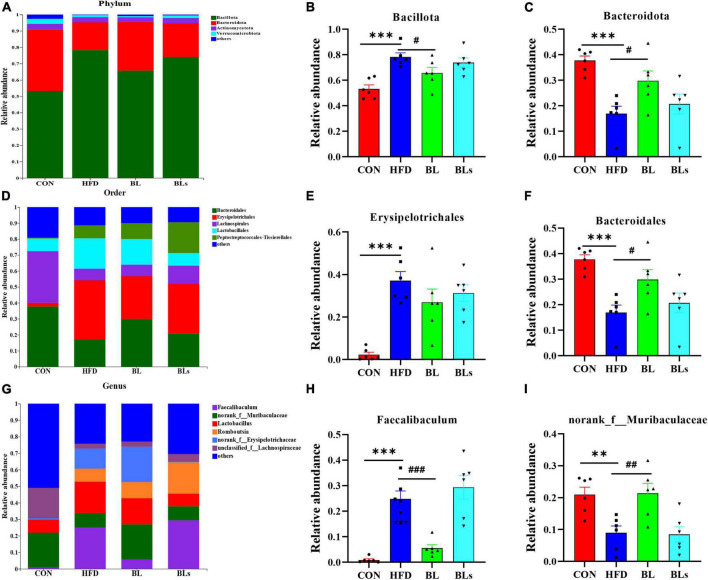
*Brevibacillus laterosporus* BL1 treatment modulated gut microbiota composition in HFD-fed mice. **(A)** Gut bacterial taxonomic profiling at the phylum level. The relative abundances of Bacillota **(B)** and Bacteroidota **(C)**. **(D)** Gut bacterial taxonomic profiles at the order level. Relative abundance of Erysipelotrichales **(E)** and Bacteroidales **(F)**. **(G)** Gut bacterial taxonomic profiling at the genus level. Relative abundances of *Faecalibaculum* **(H)** and norank_f_*Muribaculaceae* **(I)**. Data are presented as mean ± SEM (*n* = 6) and analyzed using one-way ANOVA. Significant differences between HFD and CON are indicated by **p < 0.01 and ***p < 0.001. Significant differences between HFD and BL are indicated by ^#^*p* < 0.05, ^##^*p* < 0.01, and ^###^*p* < 0.001.

To further examine the specific alterations in overall microbial communities, the dominant flora among each sample was analyzed at different taxonomic levels. The histogram illustrating gut microbiota at the phylum level revealed that the intestinal microbiota structure in all groups was occupied by Bacteroidota and Bacillota over 90% (Figure 7A). HFD administration significantly reduced Bacteroidota relative abundance and increased Bacillota relative abundance (*p* < 0.001). Nevertheless, *B. laterosporus* BL1 intervention reversed the relative abundance of these bacterial taxa in HFD-induced mice (*p* < 0.05) (Figures 7B,C). The order level analysis revealed that the HFD group possessed higher relative Erysipelotrichales levels but lower Bacteroidales levels, and *B. laterosporus* BL1 treatment significantly reversed the relative abundance of Bacteroidales (*p* < 0.05) (Figures 7D–F). Similar results were observed for *Faecalibaculum* and norank_f_*Muribaculaceae* at the genus level (Figures 7G–I). It is noteworthy that the supernatant of *B. laterosporus* BL1 intervention had no obvious effect on intestinal microbial composition in HFD-fed mice. Therefore, these data demonstrated that *B. laterosporus* BL1, but not its supernatant, can normalize the disturbance of gut microbiota induced by HFD.

### *Brevibacillus laterosporus* BL1 regulates short-chain fatty acid content in high-fat diet-fed mice

**FIGURE 8:**
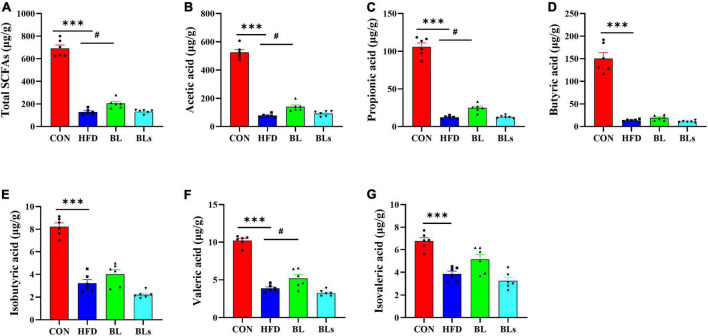
*Brevibacillus laterosporus* BL1 treatment improved SCFA concentrations in HFD-fed mice. **(A–G)** Total SCFAs, acetic acid, propionic acid, butyric acid, isobutyric acid, valeric acid, and isovaleric acid. Data are presented as mean ± SEM (*n* = 6) and analyzed using one-way ANOVA. Significant differences between HFD and CON are indicated by ***p < 0.001. Significant differences between HFD and BL are indicated by ^#^*p* < 0.05.

Increasing evidence has suggested that SCFAs, the main metabolites of dietary fiber by intestinal microbiota, regulate host lipid metabolism and intestinal microbiota. Thus, colonic SCFA contents were determined in this study. As shown in Figure 8, total SCFAs concentration was maximal in the CON group and minimal in the HFD group, and middle in the BL group. Specifically, *B. laterosporus* BL1 treatment significantly increased colonic concentrations of acetic acid, propionic acid, and valeric acid in HFD-fed mice (*p* < 0.05). However, *B. laterosporus* BL1 intervention did not significantly affect colonic butyric, isobutyric, or isovaleric acid levels in HFD-fed mice.

### Gut microbiota correlated with short-chain fatty acids and obesity-related parameters

**FIGURE 9:**
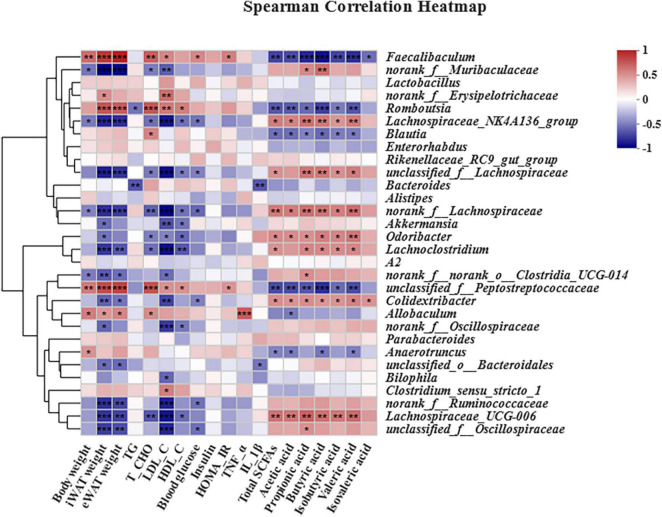
Heatmap of Spearman’s correlations between gut bacteria relative abundances and obesity-related indices. The top 30 most abundant genera in each sample were used for the hierarchical clustering and heatmap analyses based on Spearman’s correlation coefficients. The red and blue blocks represent positive and negative correlations, respectively. Significant correlations are indicated by *p < 0.05, **p < 0.01, and ***p < 0.001 (*n* = 6).

Given the amelioration of obesity-related complications and intestinal microbiota dysbiosis in HFD-induced obese mice by *B. laterosporus* BL1, Spearman’s correlation analysis was performed to identify associations between dominant gut bacterial genera and metabolites (SCFAs)/obesity-related parameters (body weight, iWAT weight, eWAT weight, TG, T-CHO, LDL-C, HDL-C, TNF-α, IL-1β, insulin, blood glucose, and HOMA-IR) (Figure 9). Most gut bacteria genera were positively correlated with SCFAs and negatively correlated with most obesity-related indices. Specifically, *Lachnospiraceae*_NK4A136_group, unclassified_f__*Lachnospiraceae*, norank_f__*Lachnospiraceae*, *Odoribacter*, *Lachnoclostridium*, *Colidextribacter*, and *Lachnospiraceae*_UCG-006 were strongly positively correlated with at least four kinds of SCFAs and strongly negatively correlated with at least four kinds of obesity-related parameters. Conversely, *Faecalibaculum*, *Romboutsia*, *Blautia*, unclassified_f__*Peptostreptococcaceae*, and *Anaerotruncus* were significantly negatively correlated with at least three SCFA indices, and positively correlated with body weight, iWAT and eWAT weight, T-CHO, LDL-C, HDL-C, blood glucose, and HOMA-IR value. Moreover, *Allobaculum* was positively correlated with TNF-α but *Bacteroides* and unclassified_o__*Bacteroidales* were negatively correlated with IL-1β.

## Discussion

Obesity has become an epidemic and a worldwide threat to public health and is responsible for the increased prevalence of chronic diseases such as atherosclerosis, diabetes, and cardiovascular diseases (38, 39). Overwhelming evidence has been reported that intestinal flora is closely associated with the development of obesity and its related metabolic diseases by influencing energy metabolism, inflammatory response, and glucose metabolism (40, 41). In recent years, increasing evidence has demonstrated that probiotics, including *Lactobacillus*, *Bifidobacterium*, and *Bacillus* can prevent obesity and its related diseases by modulation of intestinal flora in murine models and clinical trials (42–45). Thus, the regulation of gut microbiota with probiotics has attracted much attention as a potential preventive strategy against overweight and obesity. However, there are limited probiotics available as preventive agents for obesity, thus exploring novel strains with anti-obesity properties is quite necessary. Although the *B. laterosporus* BL1 strain is known for broad-spectrum antimicrobial activity, its biological activities in preventing obesity and modulating the related intestinal microbiota dysbiosis have not yet been explored. Here, the ability of *B. laterosporus* BL1 and its culture supernatant on adiposis and the underlying mechanisms associated with gut microbiota in HFD-induced obese mice were investigated. Our results found that prophylactic *B. laterosporus* BL1 bacteria treatment had protective effects against metabolic impairments and gut microbiota dysbiosis in HFD-fed mice, but these were not exerted by supernatant treatment, indicating that the bacteria rather than supernatant were responsible for the beneficial effects of *B. laterosporus* BL1. This is the first study to investigate the bioactivity of *B. laterosporus* BL1 for preventing obesity and regulating the gut microbial community. These findings indicated that *B. laterosporus* BL1 might be served as a promising probiotic to prevent obesity and its related metabolic diseases.

In HFD-fed mice, *B. laterosporus* BL1 intervention decreased body weight gain by 41.26%, which was more impactful than *Companilactobacillus* (*Lactobacillus*) *crustorum* MN047 (−29.56%) (21). Fat mass, mean size of inguinal and epididymal adipocytes, and liver lipid droplet size also significantly decreased as a result of *B. laterosporus* BL1 treatment, consistent with a previous study of the anti-obesity effects of *Bacillus licheniformis* (46). Notably, the food intake between the HFD and BL groups was not statistically significant, indicating that the beneficial effects of *B. laterosporus* BL1 on weight gain and fat accumulation were not due to a reduction in food or energy consumption. Furthermore, HFD mice presented remarkably elevated serum levels of TG, T-CHO, LDL-C, and HDL-C, while *B. laterosporus* BL1 intervention exerted an anti-hyperlipidemic effect in obese mice. Insulin resistance is one of the most common complications in obese individuals (47). As expected, our study found that *B. laterosporus* BL1 treatment significantly improved glucose tolerance and insulin resistance in HFD-induced mice, as evidenced by markedly reduced fasting blood glucose and fasting serum insulin as well as improved IGTT and ITT. Similar results were obtained in previous studies reporting that intake of a *B. licheniformis* and *B. subtilis* mixture effectively improved glucose homeostasis and conferred protection against insulin resistance (22). Therefore, these results indicated positive effects of *B. laterosporus* BL1 intervention on disorders of lipid and glucose metabolism induced by HFD.

Obesity is closely associated with chronic inflammation, which results in whole-body impaired glucose homeostasis and insulin sensitivity (48). In the setting of obesity, immune cell infiltration and proinflammatory activation increase in peripheral tissues, leading to the elevated secretion of proinflammatory cytokines. *Via* autocrine and paracrine effects of proinflammatory molecules, inflammation can interfere with whole-body insulin signaling or induce β-cell dysfunction and subsequent insulin deficiency, resulting in obesity-related insulin resistance (49, 50). In our study, *B. laterosporus* BL1 bacteria intervention remarkably decreased serum IL-1β and TNF-α levels and suppressed hepatic mRNA expression of IL-1β, TNF-α, IL-6, and IFN-γ, indicating that the amelioration of insulin resistance by *B. laterosporus* BL1 intervention may be attributable to decreases in systemic chronic inflammation. Interestingly, these anti-inflammatory effects were also observed in supernatant-treated mice, which is consistent with previous studies showing that *Lacticaseibacillus* (*Lactobacillus*) *rhamnosus* GG culture supernatant significantly inhibited hepatic inflammation and liver injury by attenuation of TNFα production in mice with fatty livers (51).

Accumulating evidence has demonstrated that HFD-induced obesity may alter the expression of lipid-related genes in adipose tissue and the liver such as SREBP1, PPARγ, FAS, CD36, HSL, and CPT-1 (52, 53). Some probiotics regulate the expression of genes related to lipogenesis and lipid metabolism to prevent obesity and ameliorate serum lipid levels (21, 46, 54). In general, SREBP1 is a key transcription factor regulating the expression of multiple lipases which participate in adipocyte differentiation and adipogenesis and then catalyze fatty acid and TG synthesis (55, 56). PPARγ, a well-known nuclear receptor, plays a crucial role in fatty acid uptake and lipogenesis, and down-regulated PPARγ may inhibit downstream enzymes catalyzing fatty acid and TG synthesis, such as FAS (23, 57). CD36 is a multifunctional membrane protein that is critical to facilitating the absorption and intracellular transport of long-chain fatty acids. Furthermore, CD36 is involved in the regulation of immune responses, chronic metabolic inflammation, angiogenesis, and atherogenesis (58, 59). HSL is one of the key rate-limiting enzymes for lipolysis initiation and catalyzes TG hydrolysis to generate free fatty acid and glycerol (60, 61). CPT-1, the first rate-limiting enzyme in fatty acid β-oxidation, catalyzes the transfer of long-chain fatty acyl-CoA from coenzyme A to L-carnitine and ultimately promotes mitochondrial fatty acid oxidation (23, 62). In the present study, *B. laterosporus* BL1 treatment significantly decreased the expression of SREBP1, PPARγ, FAS, and CD36, as well as increased the expression of HSL and CPT-1 in the liver. Moreover, the expression of FAS and CD36 significantly decreased in the iWAT following *B. laterosporus* BL1 treatment, and HSL expression increased. In addition, BAT is a potential preventive target for obesity, and its activation can stimulate thermogenesis and energy expenditure, thus increasing body weight loss and potentially lowering adipose tissue inflammation (63, 64). Our present study revealed that *B. laterosporus* BL1 supplementation prevented HFD-induced whitening of BAT and increased its activity, as manifested by decreased mean adipocyte size, increased thermogenesis of BAT, and upregulated expression of key genes related to BAT activation (e.g., UCP1, CPT-1, PGC-1α, and PRDM16). Similarly, *Dysosmobacter welbionis*, a human commensal bacterium, has been reported to promote BAT activation by elevated mitochondria number and non-shivering thermogenesis (65). Therefore, these results suggested that *B. laterosporus* BL1 treatment may prevent HFD-induced obesity by reducing fat lipogenesis and accumulation as well as enhancing β-oxidation and BAT activation.

Accumulating evidence suggests that obesity-induced metabolic disorder is closely correlated with intestinal microbiota dysbiosis (29, 66). As a crucial environmental factor and a novel therapeutic target for obesity-related metabolic syndrome, the gut microbiota has been explored deeply in recent years. Moreover, several studies have reported that the beneficial effects of probiotics on HFD-induced obesity and metabolic disorder are mediated by intestinal microbiota (11, 12, 45). It is well-documented that the microbial diversity of healthy-weight individuals is higher than that of obese individuals (67). Consistent with a previous study (68), our study showed that HFD treatment significantly altered gut microbiota richness and abundance based on the results of α-diversity and Venn diagrams analyses. Although *B. laterosporus* BL1 treatment did not significantly change gut microbiota α-diversity, a distinct clustering pattern in gut microbiota structure between the HFD and BL groups was observed. In addition, HFD treatment may result in increased Bacillota relative abundance and decreased Bacteroidota relative abundance (28). Bacillota and Bacteroidota are the dominant phyla in human intestinal microbiota, occupying over 90% of all sequences, and are mainly responsible for energy absorption related to gut flora (36, 69). In the current study, *B. laterosporus* BL1 intervention significantly reversed the relative abundance of Bacillota and Bacteroidota. This is similar to the findings that the prevention of *B. amyloliquefaciens* SC06 in HFD-induced obesity is accompanied by a reduction in Bacillota relative abundance and an elevation in Bacteroidota relative abundance (10). Collectively, our results suggested that *B. laterosporus* BL1 intervention helped maintain a relatively healthy microbiome, thus preventing obesity in HFD-fed mice.

Our results showed that *B. laterosporus* BL1 administration also significantly reversed some of the alterations in gut microbiota induced by an HFD, including Bacteroidales at the order level, and *Faecalibaculum* and norank_f_*Muribaculaceae* at the genus level. Bacteroidales plays a key role in the host metabolism of carbohydrates and proteins associated with gut flora, which is negatively correlated with body weight gain and fat accumulation (70). A rodent model showed that the elevation of Bacteroidales abundance by oligosaccharides from *Gracilaria lemaneiformis* may help attenuate HFD-induced obesity and metabolic syndrome (71). *Faecalibaculum* is a proinflammatory bacterium that may impair the gut barrier and is relative to various metabolic diseases, such as obesity, cardiovascular disease, and diabetes (72). Conversely, norank_f_*Muribaculaceae* is a potentially beneficial bacterium, and elevated norank_f_*Muribaculaceae* concentration helped to alleviate obesity and insulin resistance in HFD-induced mice (73). These dominant bacterial taxa, which were reversed by *B. laterosporus* BL1 intervention, may have helped prevent obesity and metabolic syndrome in the current study. Furthermore, Spearman’s correlation analysis revealed that the bacteria that increased in abundance as a result of HFD administration were positively correlated with obesity-related parameters but negatively correlated with SCFAs concentration. On the contrary, *B. laterosporus* BL1 treatment promoted expansion in the relative abundance of bacterial genera that were positively correlated with SCFAs but negatively correlated with obesity-related parameters (e.g., norank_f_*Muribaculaceae*, *Odoribacter*, and *Lachnoclostridium*). Notably, our study found that SCFAs, the important signaling molecules in the communication between host and gut microbiota (74), were significantly increased by *B. laterosporus* BL1 treatment, especially acetic acid, propionic acid, and valeric acid. These results suggested that another possible mechanism of action of *B. laterosporus* BL1 preventing obesity was the promotion of SCFAs-producing bacteria and the subsequent elevation of colonic SCFA concentrations, resulting in increased body weight loss in HFD-fed mice.

In conclusion, this study suggested that prophylactic *B. laterosporus* BL1 bacteria supplementation effectively prevented body weight gain, fat accumulation, chronic inflammation, and BAT whitening, as well as attenuated lipid profiles and insulin resistance in HFD-induced mice. *B. laterosporus* BL1 bacteria intervention may also modulate the HFD-induced structural and componential alteration of gut flora, especially by increasing the relative abundance of SCFA-producing bacteria and subsequently elevating SCFA levels, which may be associated with the role of *B. laterosporus* BL1 in preventing obesity. Our study demonstrated that *B. laterosporus* BL1 is a promising probiotic candidate to prevent obesity and its related syndrome. However, HFD is characterized by a high-fat content and monotonicity compared to the human diet, this may affect the credibility and translational value of experiment results (75). Therefore, further investigations, especially long-term clinical trials, are required to confirm these findings.

## Data availability statement

The datasets presented in this study can be found in online repositories. The names of the repository/repositories and accession number(s) can be found below: https://www.ncbi.nlm.nih.gov/, PRJNA876212.

## Ethics statement

This animal study was reviewed and approved by the Animal Care and Use Committee of South China Agricultural University.

## Author contributions

GW and JH participated in the writing and editing of the manuscript. XM, MS, and YY contributed to the study design. DD and JD revised the manuscript. All authors contributed to the article and approved the submitted version.

## References

[1] Dietz WH Baur LA Hall K Puhl RM Taveras EM Uauy R Management of obesity: improvement of health-care training and systems for prevention and care. *Lancet.* (2015) 385:2521–33. 10.1016/s0140-6736(14)61748-7 25703112

[2] Tang C Kong L Shan M Lu Z Lu Y. Protective and ameliorating effects of probiotics against diet-induced obesity: a review. *Food Res Int.* (2021) 147:110490. 10.1016/j.foodres.2021.110490 34399486

[3] Shin NR Lee JC Lee HY Kim MS Whon TW Lee MS An increase in the *Akkermansia* spp. population induced by metformin treatment improves glucose homeostasis in diet-induced obese mice. *Gut.* (2014) 63:727–35. 10.1136/gutjnl-2012-303839 23804561

[4] Perry RJ Samuel VT Petersen KF Shulman GI. The role of hepatic lipids in hepatic insulin resistance and type 2 diabetes. *Nature.* (2014) 510:84–91. 10.1038/nature13478 24899308 PMC4489847

[5] Ormazabal V Nair S Elfeky O Aguayo C Salomon C Zuñiga FA. Association between insulin resistance and the development of cardiovascular disease. *Cardiovasc Diabetol.* (2018) 17:122. 10.1186/s12933-018-0762-4 30170598 PMC6119242

[6] Weng G Duan Y Zhong Y Song B Zheng J Zhang S Plant extracts in obesity: a role of gut microbiota. *Front Nutr.* (2021) 8:727951. 10.3389/fnut.2021.727951 34631766 PMC8495072

[7] Gadde KM Martin CK Berthoud HR Heymsfield SB. Obesity: pathophysiology and management. *J Am Coll Cardiol.* (2018) 71:69–84. 10.1016/j.jacc.2017.11.011 29301630 PMC7958889

[8] Siebenhofer A Jeitler K Horvath K Berghold A Posch N Meschik J Long-term effects of weight-reducing drugs in people with hypertension. *Cochrane Database Syst Rev.* (2016) 3:CD007654. 10.1002/14651858.CD007654.pub4 26934640

[9] Hill C Guarner F Reid G Gibson GR Merenstein DJ Pot B Expert consensus document. The International Scientific Association for Probiotics and Prebiotics consensus statement on the scope and appropriate use of the term probiotic. *Nat Rev Gastroenterol Hepatol.* (2014) 11:506–14. 10.1038/nrgastro.2014.66 24912386

[10] Wang Y Wu Y Wang B Xu H Mei X Xu X *Bacillus amyloliquefaciens* SC06 protects mice against high-fat diet-induced obesity and liver injury via regulating host metabolism and gut microbiota. *Front Microbiol.* (2019) 10:1161. 10.3389/fmicb.2019.01161 31191487 PMC6547872

[11] Liu Y Gao Y Ma F Sun M Mu G Tuo Y. The ameliorative effect of *Lactobacillus* plantarum Y44 oral administration on inflammation and lipid metabolism in obese mice fed with a high fat diet. *Food Funct.* (2020) 11:5024–39. 10.1039/d0fo00439a 32530448

[12] Kim B Park KY Ji Y Park S Holzapfel W Hyun CK. Protective effects of *Lactobacillus* rhamnosus GG against dyslipidemia in high-fat diet-induced obese mice. *Biochem Biophys Res Commun.* (2016) 473:530–6. 10.1016/j.bbrc.2016.03.107 27018382

[13] Molina-Tijeras JA Diez-Echave P Vezza T Hidalgo-Garcia L Ruiz-Malagon AJ Rodriguez-Sojo MJ *Lactobacillus* fermentum CECT5716 ameliorates high fat diet-induced obesity in mice through modulation of gut microbiota dysbiosis. *Pharmacol Res.* (2021) 167:105471. 10.1016/j.phrs.2021.105471 33529749

[14] Kondo S Xiao JZ Satoh T Odamaki T Takahashi S Sugahara H Antiobesity effects of *Bifidobacterium* breve strain B-3 supplementation in a mouse model with high-fat diet-induced obesity. *Biosci Biotechnol Biochem.* (2010) 74:1656–61. 10.1271/bbb.100267 20699581

[15] Tenorio-Jimenez C Martinez-Ramirez MJ Gil A Gomez-Llorente C. Effects of probiotics on metabolic syndrome: a systematic review of randomized clinical trials. *Nutrients.* (2020) 12:124. 10.3390/nu12010124 31906372 PMC7019472

[16] Dai K Song Y Zhang D Wei Y Jiang S Xu F Thinned peach polyphenols alleviate obesity in high fat mice by affecting gut microbiota. *Food Res Int.* (2022) 157:111255. 10.1016/j.foodres.2022.111255 35761567

[17] Li S-Z Zeng S-L Liu EH. Anti-obesity natural products and gut microbiota. *Food Res Int.* (2022) 151:110819. 10.1016/j.foodres.2021.110819 34980371

[18] Turnbaugh PJ Ridaura VK Faith JJ Rey FE Knight R Gordon JI. The effect of diet on the human gut microbiome: a metagenomic analysis in humanized gnotobiotic mice. *Sci Transl Med.* (2009) 1:6ra14. 10.1126/scitranslmed.3000322 20368178 PMC2894525

[19] Martinez-Guryn K Hubert N Frazier K Urlass S Musch MW Ojeda P Small intestine microbiota regulate host digestive and absorptive adaptive responses to dietary lipids. *Cell Host Microbe.* (2018) 23:458–69.e5. 10.1016/j.chom.2018.03.011 29649441 PMC5912695

[20] David LA Maurice CF Carmody RN Gootenberg DB Button JE Wolfe BE Diet rapidly and reproducibly alters the human gut microbiome. *Nature.* (2014) 505:559–63. 10.1038/nature12820 24336217 PMC3957428

[21] Wang T Yan H Lu Y Li X Wang X Shan Y Anti-obesity effect of *Lactobacillus rhamnosus* LS-8 and *Lactobacillus* crustorum MN047 on high-fat and high-fructose diet mice base on inflammatory response alleviation and gut microbiota regulation. *Eur J Nutr.* (2020) 59:2709–28. 10.1007/s00394-019-02117-y 31659451

[22] Cao GT Dai B Wang KL Yan Y Xu YL Wang YX *Bacillus licheniformis*, a potential probiotic, inhibits obesity by modulating colonic microflora in C57BL/6J mice model. *J Appl Microbiol.* (2019) 127:880–8. 10.1111/jam.14352 31211897

[23] Chen G Xie M Wan P Chen D Dai Z Ye H Fuzhuan brick tea polysaccharides attenuate metabolic syndrome in high-fat diet induced mice in association with modulation in the gut microbiota. *J Agric Food Chem.* (2018) 66:2783–95. 10.1021/acs.jafc.8b00296 29514453

[24] Lim J-J Jung AH Joo Suh H Choi H-S Kim H. *Lactiplantibacillus plantarum* K8-based paraprobiotics prevents obesity and obesity-induced inflammatory responses in high fat diet-fed mice. *Food Res Int.* (2022) 155:111066. 10.1016/j.foodres.2022.111066 35400444

[25] Ruiu L. *Brevibacillus* laterosporus, a pathogen of invertebrates and a broad-spectrum antimicrobial species. *Insects.* (2013) 4:476–92. 10.3390/insects4030476 26462431 PMC4553477

[26] Barbieri G Ferrari C Mamberti S Gabrieli P Castelli M Sassera D Identification of a novel strain with insecticidal activity against larvae. *Front Microbiol.* (2021) 12:624014. 10.3389/fmicb.2021.624014 33679643 PMC7925996

[27] Ruiu L Satta A Floris I. Administration of *Brevibacillus* laterosporus spores as a poultry feed additive to inhibit house fly development in feces: a new eco-sustainable concept. *Poult Sci.* (2014) 93:519–26. 10.3382/ps.2013-03418 24604843

[28] Duan Y Zhong Y Xiao H Zheng C Song B Wang W Gut microbiota mediates the protective effects of dietary beta-hydroxy-beta-methylbutyrate (HMB) against obesity induced by high-fat diets. *FASEB J.* (2019) 33:10019–33. 10.1096/fj.201900665RR 31167080

[29] Yin J Li Y Han H Chen S Gao J Liu G Melatonin reprogramming of gut microbiota improves lipid dysmetabolism in high-fat diet-fed mice. *J Pineal Res.* (2018) 65:e12524. 10.1111/jpi.12524 30230594

[30] Yu M Li Z Rong T Wang G Liu Z Chen W Different dietary starch sources alter the carcass traits, meat quality, and the profile of muscle amino acid and fatty acid in finishing pigs. *J Anim Sci Biotechnol.* (2020) 11:78. 10.1186/s40104-020-00484-9 32782789 PMC7412799

[31] Tian Z Cui Y Lu H Wang G Ma X. Effect of long-term dietary probiotic *Lactobacillus* reuteri 1 or antibiotics on meat quality, muscular amino acids and fatty acids in pigs. *Meat Sci.* (2021) 171:108234. 10.1016/j.meatsci.2020.108234 32906013

[32] Tang Q Yi H Hong W Wu Q Yang X Hu S Comparative effects of *L. plantarum* CGMCC 1258 and *L. reuteri* LR1 on growth performance, antioxidant function, and intestinal immunity in weaned pigs. *Front Vet Sci.* (2021) 8:728849. 10.3389/fvets.2021.728849 34859082 PMC8632148

[33] Chen S Zhou Y Chen Y Gu J. Fastp: an ultra-fast all-in-one FASTQ preprocessor. *Bioinformatics.* (2018) 34:i884–90. 10.1093/bioinformatics/bty560 30423086 PMC6129281

[34] Magoc T Salzberg SL. FLASH: fast length adjustment of short reads to improve genome assemblies. *Bioinformatics.* (2011) 27:2957–63. 10.1093/bioinformatics/btr507 21903629 PMC3198573

[35] Yang K Deng X Jian S Zhang M Wen C Xin Z Gallic acid alleviates gut dysfunction and boosts immune and antioxidant activities in puppies under environmental stress based on microbiome-metabolomics analysis. *Front Immunol.* (2021) 12:813890. 10.3389/fimmu.2021.813890 35095912 PMC8795593

[36] Zhang XY Chen J Yi K Peng L Xie J Gou X Phlorizin ameliorates obesity-associated endotoxemia and insulin resistance in high-fat diet-fed mice by targeting the gut microbiota and intestinal barrier integrity. *Gut Microbes.* (2020) 12:1–18. 10.1080/19490976.2020.1842990 33222603 PMC7714487

[37] Gregor MF Hotamisligil GS. Inflammatory mechanisms in obesity. *Annu Rev Immunol.* (2011) 29:415–45. 10.1146/annurev-immunol-031210-101322 21219177

[38] Piché M-E Tchernof A Després J-P. Obesity phenotypes, diabetes, and cardiovascular diseases. *Circ Res.* (2020) 126:1477–500. 10.1161/CIRCRESAHA.120.316101 32437302

[39] Conway B Rene A. Obesity as a disease: no lightweight matter. *Obes Rev.* (2004) 5:145–51. 10.1111/j.1467-789X.2004.00144.x 15245383

[40] Torres-Fuentes C Schellekens H Dinan TG Cryan JF. The microbiota-gut-brain axis in obesity. *Lancet Gastroenterol Hepatol.* (2017) 2:747–56. 10.1016/S2468-1253(17)30147-4 28844808

[41] Gérard P. Gut microbiota and obesity. *Cell Mol Life Sci.* (2016) 73:147–62. 10.1007/s00018-015-2061-5 26459447 PMC11108539

[42] Crovesy L Ostrowski M Ferreira D Rosado EL Soares-Mota M. Effect of *Lactobacillus* on body weight and body fat in overweight subjects: a systematic review of randomized controlled clinical trials. *Int J Obes.* (2017) 41:1607–14. 10.1038/ijo.2017.161 28792488

[43] Song W Song C Li L Wang T Hu J Zhu L *Lactobacillus* alleviated obesity induced by high-fat diet in mice. *J Food Sci.* (2021) 86:5439–51. 10.1111/1750-3841.15971 34859434

[44] Sergeev IN Aljutaily T Walton G Huarte E. Effects of synbiotic supplement on human gut microbiota, body composition and weight loss in obesity. *Nutrients.* (2020) 12:222. 10.3390/nu12010222 31952249 PMC7019807

[45] Zeng Z Zhou Y Xu Y Wang S Wang B Zeng Z *Bacillus* amyloliquefaciens SC06 alleviates the obesity of ob/ob mice and improves their intestinal microbiota and bile acid metabolism. *Food Funct.* (2022) 13:5381–95. 10.1039/d1fo03170h 35470823

[46] Lu X Jing Y Zhou X Zhang N Tai J Cao Y. *Bacillus* licheniformis zhengchangsheng(R) inhibits obesity by regulating the AMP-activated protein kinase signaling pathway. *Probiotics Antimicrob Proteins.* (2021) 13:1658–67. 10.1007/s12602-021-09792-6 33954883

[47] Johnson JD. On the causal relationships between hyperinsulinaemia, insulin resistance, obesity and dysglycaemia in type 2 diabetes. *Diabetologia.* (2021) 64:2138–46. 10.1007/s00125-021-05505-4 34296322

[48] Samad F Ruf W. Inflammation, obesity, and thrombosis. *Blood.* (2013) 122:3415–22. 10.1182/blood-2013-05-427708 24092932 PMC3829115

[49] Wu H Ballantyne CM. Skeletal muscle inflammation and insulin resistance in obesity. *J Clin Invest.* (2017) 127:43–54. 10.1172/JCI88880 28045398 PMC5199705

[50] Esser N Legrand-Poels S Piette J Scheen AJ Paquot N. Inflammation as a link between obesity, metabolic syndrome and type 2 diabetes. *Diabet Res Clin Pract.* (2014) 105:141–50. 10.1016/j.diabres.2014.04.006 24798950

[51] Wang Y Liu Y Kirpich I Ma Z Wang C Zhang M *Lactobacillus* rhamnosus GG reduces hepatic TNFalpha production and inflammation in chronic alcohol-induced liver injury. *J Nutr Biochem.* (2013) 24:1609–15. 10.1016/j.jnutbio.2013.02.001 23618528 PMC3804118

[52] Zhong Y Song B Zheng C Zhang S Yan Z Tang Z Flavonoids from mulberry leaves alleviate lipid dysmetabolism in high fat diet-fed mice: involvement of gut microbiota. *Microorganisms.* (2020) 8:860. 10.3390/microorganisms8060860 32517288 PMC7355566

[53] Song B Zhong YZ Zheng CB Li FN Duan YH Deng JP. Propionate alleviates high-fat diet-induced lipid dysmetabolism by modulating gut microbiota in mice. *J Appl Microbiol.* (2019) 127:1546–55. 10.1111/jam.14389 31325215

[54] Kim B Kwon J Kim MS Park H Ji Y Holzapfel W Protective effects of *Bacillus* probiotics against high-fat diet-induced metabolic disorders in mice. *PLoS One.* (2018) 13:e0210120. 10.1371/journal.pone.0210120 30596786 PMC6312313

[55] Chen G Xie M Dai Z Wan P Ye H Zeng X Kudingcha and fuzhuan brick tea prevent obesity and modulate gut microbiota in high-fat diet fed mice. *Mol Nutr Food Res.* (2018) 62:e1700485. 10.1002/mnfr.201700485 29345748

[56] Hu WY Ma XH Zhou WY Li XX Sun TT Sun H. Preventive effect of Silibinin in combination with Pu-erh tea extract on non-alcoholic fatty liver disease in ob/ob mice. *Food Funct.* (2017) 8:1105–15. 10.1039/c6fo01591c 28164196

[57] Ye J Zhao Y Chen X Zhou H Yang Y Zhang X Pu-erh tea ameliorates obesity and modulates gut microbiota in high fat diet fed mice. *Food Res Int.* (2021) 144:110360. 10.1016/j.foodres.2021.110360 34053553

[58] Zhao L Zhang C Luo X Wang P Zhou W Zhong S CD36 palmitoylation disrupts free fatty acid metabolism and promotes tissue inflammation in non-alcoholic steatohepatitis. *J Hepatol.* (2018) 69:705–17. 10.1016/j.jhep.2018.04.006 29705240

[59] Pepino MY Kuda O Samovski D Abumrad NA. Structure-function of CD36 and importance of fatty acid signal transduction in fat metabolism. *Annu Rev Nutr.* (2014) 34:281–303. 10.1146/annurev-nutr-071812-161220 24850384 PMC4329921

[60] Bolsoni-Lopes A Alonso-Vale MI. Lipolysis and lipases in white adipose tissue - an update. *Arch Endocrinol Metab.* (2015) 59:335–42. 10.1590/2359-3997000000067 26331321

[61] Fruhbeck G Mendez-Gimenez L Fernandez-Formoso JA Fernandez S Rodriguez A. Regulation of adipocyte lipolysis. *Nutr Res Rev.* (2014) 27:63–93. 10.1017/S095442241400002X 24872083

[62] Joshi PR Zierz S. Muscle carnitine palmitoyltransferase II (CPT II) deficiency: a conceptual approach. *Molecules.* (2020) 25:1784. 10.3390/molecules25081784 32295037 PMC7221885

[63] Van Den Berg SM Van Dam AD Rensen PC De Winther MP Lutgens E. Immune modulation of brown(ing) adipose tissue in obesity. *Endocr Rev.* (2017) 38:46–68. 10.1210/er.2016-1066 27849358

[64] Marlatt KL Ravussin E. Brown adipose tissue: an update on recent findings. *Curr Obes Rep.* (2017) 6:389–96. 10.1007/s13679-017-0283-6 29101739 PMC5777285

[65] Le Roy T Moens De Hase E Van Hul M Paquot A Pelicaen R Regnier M Dysosmobacter welbionis is a newly isolated human commensal bacterium preventing diet-induced obesity and metabolic disorders in mice. *Gut.* (2022) 71:534–43. 10.1136/gutjnl-2020-323778 34108237 PMC8862106

[66] Kang C Wang B Kaliannan K Wang X Lang H Hui S Gut microbiota mediates the protective effects of dietary capsaicin against chronic low-grade inflammation and associated obesity induced by high-fat diet. *mBio.* (2017) 8:e00470–7. 10.1128/mBio.00470-17 28536285 PMC5442453

[67] Yang H Xiang Y Robinson K Wang J Zhang G Zhao J Gut microbiota is a major contributor to adiposity in pigs. *Front Microbiol.* (2018) 9:3045. 10.3389/fmicb.2018.03045 30619136 PMC6296290

[68] Zhong H Wang J Abdullah Hafeez MA Guan R Feng F. *Lactobacillus plantarum* ZJUFB2 prevents high fat diet-induced insulin resistance in association with modulation of the gut microbiota. *Front Nutr.* (2021) 8:754222. 10.3389/fnut.2021.754222 34805244 PMC8604096

[69] Isaacson R Kim HB. The intestinal microbiome of the pig. *Anim Health Res Rev.* (2012) 13:100–9. 10.1017/S1466252312000084 22853934

[70] Jiang P Zheng W Sun X Jiang G Wu S Xu Y Sulfated polysaccharides from *Undaria pinnatifida* improved high fat diet-induced metabolic syndrome, gut microbiota dysbiosis and inflammation in BALB/c mice. *Int J Biol Macromol.* (2021) 167:1587–97. 10.1016/j.ijbiomac.2020.11.116 33217459

[71] Xu Y Ai C Jiang P Sun X Liu Y Jiang G Oligosaccharides from *Gracilaria* lemaneiformis better attenuated high fat diet-induced metabolic syndrome by promoting the Bacteroidales proliferation. *Food Funct.* (2020) 11:1049–62. 10.1039/c9fo01996k 31819936

[72] Cai W Xu J Li G Liu T Guo X Wang H Ethanol extract of propolis prevents high-fat diet-induced insulin resistance and obesity in association with modulation of gut microbiota in mice. *Food Res Int.* (2020) 130:108939. 10.1016/j.foodres.2019.108939 32156386

[73] Zhao Q Hou D Fu Y Xue Y Guan X Shen Q. Adzuki bean alleviates obesity and insulin resistance induced by a high-fat diet and modulates gut microbiota in mice. *Nutrients.* (2021) 13:3240. 10.3390/nu13093240 34579118 PMC8466346

[74] Zheng W Duan M Jia J Song S Ai C. Low-molecular alginate improved diet-induced obesity and metabolic syndrome through modulating the gut microbiota in BALB/c mice. *Int J Biol Macromol.* (2021) 187:811–20. 10.1016/j.ijbiomac.2021.08.003 34363822

[75] Kleinert M Clemmensen C Hofmann SM Moore MC Renner S Woods SC Animal models of obesity and diabetes mellitus. *Nat Rev Endocrinol.* (2018) 14:140–62. 10.1038/nrendo.2017.161 29348476

