# Supplementary material for: Brevibacillus laterosporus BL1, a promising probiotic, prevents obesity and modulates gut microbiota in mice fed a high-fat diet
Source: Front Nutr. 2022 Nov 24;9:1050025. doi: 10.3389/fnut.2022.1050025 (PMC9729748; doi:10.3389/fnut.2022.1050025)
Supplement: Supplementary file 1 [file Data_Sheet_1.docx]

**Supplementary Fig. 1**

**
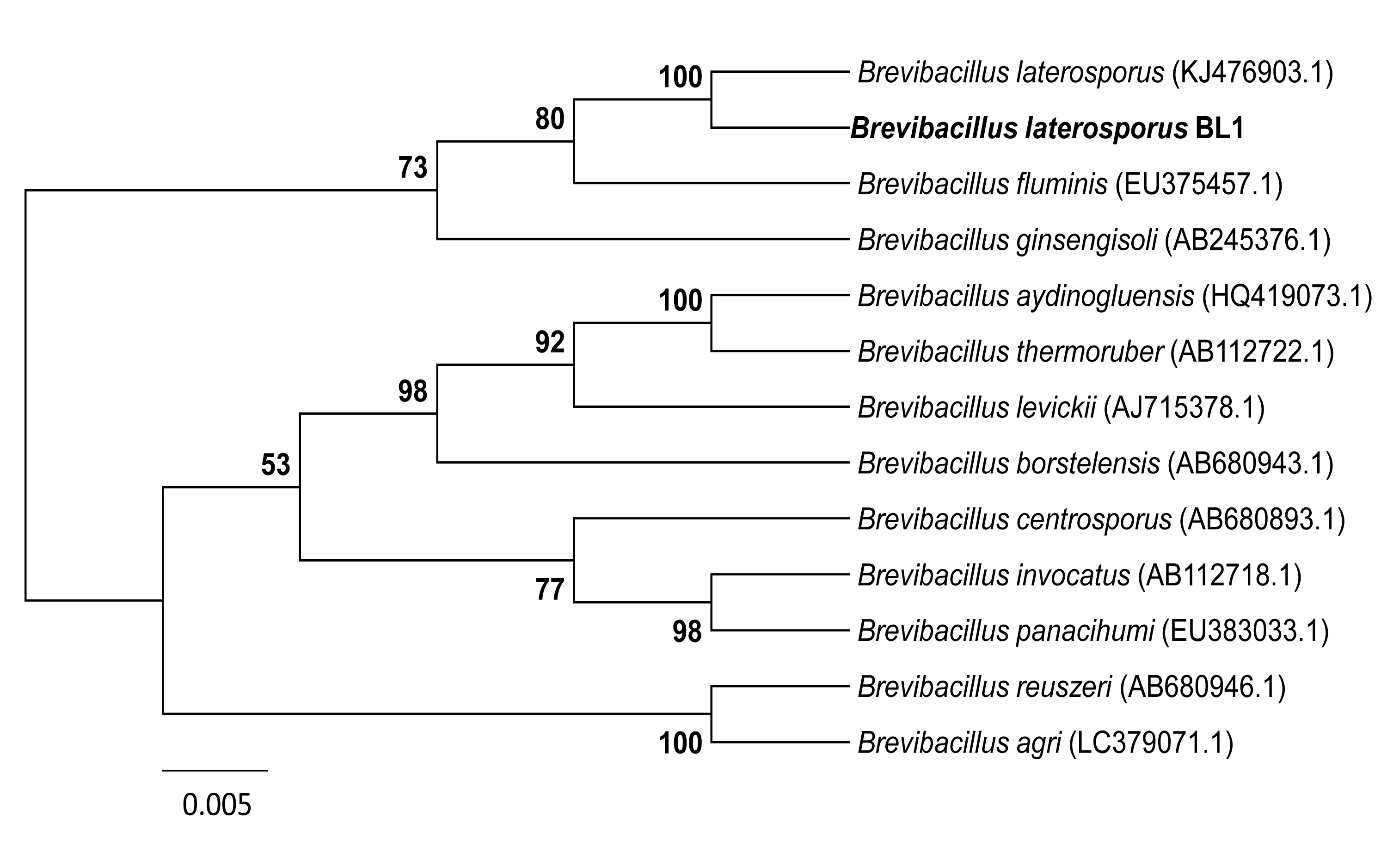
**

**Supplementary Fig. 1** Neighbor-joining phylogenetic tree of BL1 is constructed based on 16S rRNA gene sequences. The phylogenetic tree shows the relationships between individual isolates and species within the respective genus. Bootstrap values（expressed as percentages of 1000 replications）given at nodes are > 50%. Bar 0.005% sequence variation. GenBank accession numbers are given in parentheses.

**Supplementary Table 1** Composition and nutrient levels of diets.

| **Product #** | **XTCON50J** | | **XTHF60** | |
| --- | --- | --- | --- | --- |
| **Energy and nutrient composition** | gm% | kcal% | gm% | kcal% |
| Protein | 19.2% | 20% | 26% | 20% |
| Carbohydrate | 67.3% | 70% | 26% | 20% |
| Fat | 4.3% | 10% | 35% | 60% |
| Total |  | 100 |  | 100 |
| kcal/gm | 3.85 |  | 5.24 |  |
| **Ingredient** | gm | kcal | gm | kcal |
| Casein | 200 | 800 | 200 | 800 |
| L-Cystine | 3 | 12 | 3 | 12 |
| Corn Starch | 506.2 | 2024.8 | 0 | 0 |
| Maltodextrin | 125 | 500 | 125 | 500 |
| Sucrose | 68.8 | 275 | 68.8 | 275 |
| Cellulose, BW200 | 50 | 0 | 50 | 0 |
| Soybean Oil | 25 | 225 | 25 | 225 |
| Lard | 20 | 180 | 245 | 2205 |
| Mineral Mix S10026 | 10 | 0 | 10 | 0 |
| DiCalcium Phosphate | 13 | 0 | 13 | 0 |
| Calcium Carbonate | 5.5 | 0 | 5.5 | 0 |
| Potassium Citrate, 1 H2O | 16.5 | 0 | 16.5 | 0 |
| Vitamin Mix V10001 | 10 | 40 | 10 | 40 |
| Choline Bitartrate | 2 | 0 | 2 | 0 |
| FD&C Yellow Dye #5 | 0.4 | 0 | 0 | 0 |
| FD&C Red Dye #40 | 0 | 0 | 0 | 0 |
| FD&C Blue Dye #1 | 0.01 | 0 | 0.05 | 0 |
| Total | 1055.41 | 4057 | 773.85 | 4057 |

| Gene | Nucleotide sequence (5-3′) |
| --- | --- |
| SREBP1 | F: GAACGACATCGAAGACATGC |
|  | R: GAGAAGCTCTCAGGAGAG |
| PPARγ | F: GTACTGTCGGTTTCAGAAGTGCC |
|  | R: ATCTCCGCCAACAGCTTCTCCT |
| FAS | F: TTGCTGTCAACCATGCCAACCTG |
|  | R: CCATGCTCTTCATCGCAGAGTGTG |
| CD36 | F: GGAGTGGTGATGTTTGTTGCT |
|  | R: GCACACACCACCATTTCTTCT |
| HSL | F: CCAGCCTGAGGGCTTACTG |
|  | R: CTCCATTGACTGTGACATCTCG |
| CPT-1 | F: ATGTATCGCCGCAAACTGGACC |
|  | R: CTCTGAGAGGTGCTGTAGCAAG |
| UCP1 | F: GCTTTGCCTCACTCAGGATTGG |
|  | R: CCAATGAACACTGCCACACCTC |
| PGC-1α | F: AGCCGTGACCACTGACAACGAG |
|  | R: GCTGCATGGTTCTGAGTGCTAAG |
| C/EBPα | F: AGAAGTCGGTGGACAAGAACA |
|  | R: TTTGGCTTTATCTCGGCTCT |
| PRDM16 | F: GGCGAGGAAGCTAGCCAAA |
|  | R: GGTCTCCTCCTCGGCACTCT |
| Il-1β | F: GAAATGCCACCTTTTGACAGTG |
|  | R: TGGATGCTCTCATCAGGACAG |
| TNF-α | F: CTGAACTTCGGGGTGATCGG |
|  | R: GGCTTGTCACTCGAATTTTGAGA |
| Il-6 | F: TAGTCCTTCCTACCCCAATTTCC |
|  | R: TTGGTCCTTAGCCACTCCTTC |
| IFN-γ | F: CAGGCCATCAGCAACAACATAAG |
|  | R: AGCTGGTGGACCACTCGGATG |
| β-actin | F: GTCCCTCACCCTCCCAAAAG |
|  | R: GCTGCCTCAACACCTCAACCC |

**Supplementary Table 2** Primers used in this study.

**Supplementary Fig. 2**

**
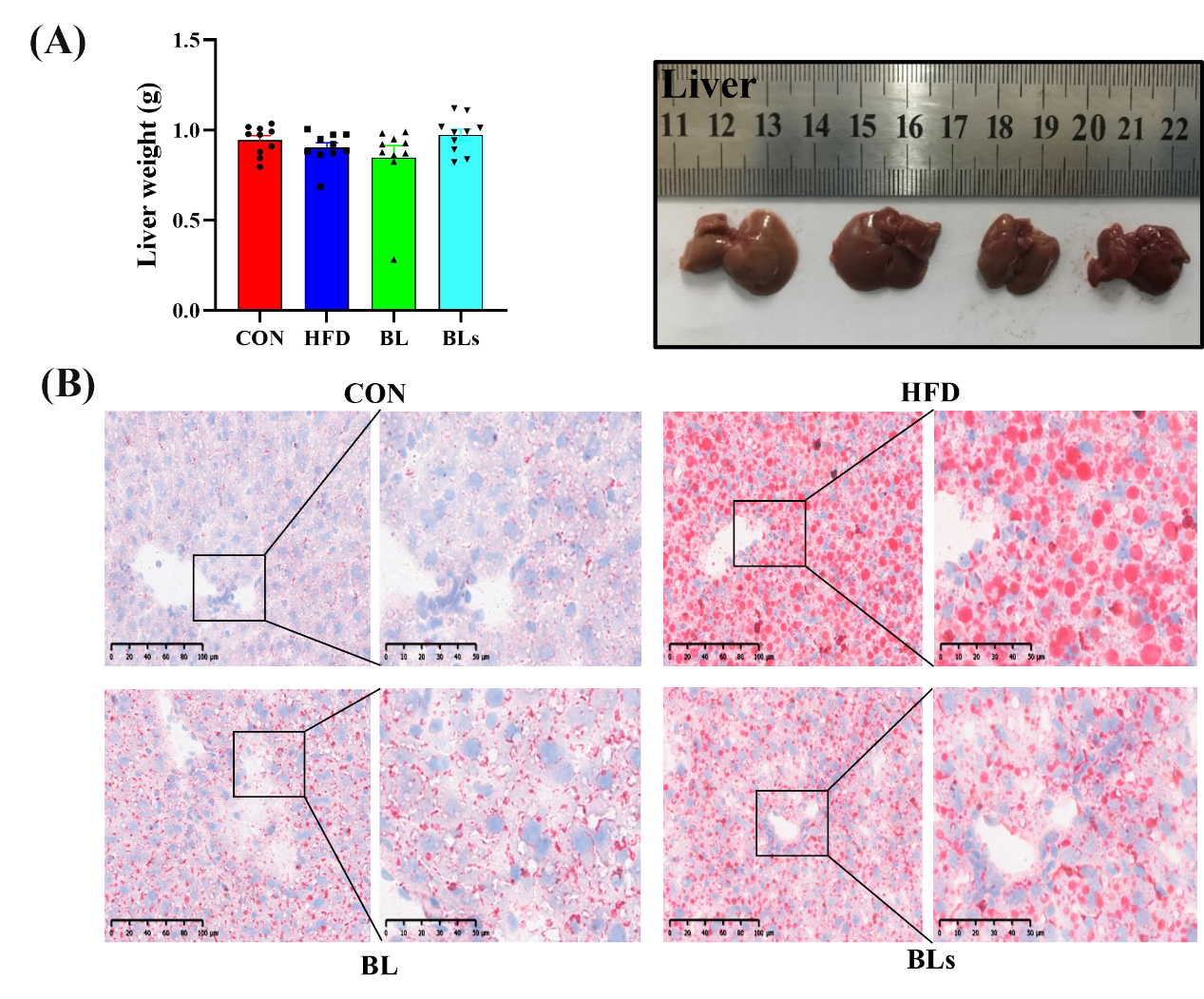
**

**Supplementary Fig. 2** Effects of *B. Laterosporus* BL1 on liver weight and lipid accumulation in HFD-fed mice. (A) Liver weight and representative liver, (B) liver oil red O staining, scale bar = 50 or 100 μm. Data are presented as mean ± SEM (n = 10 for liver weight; n = 6 for liver oil red O staining) and analyzed using one-way ANOVA and no significant difference was observed.

**Supplementary Fig. 3**


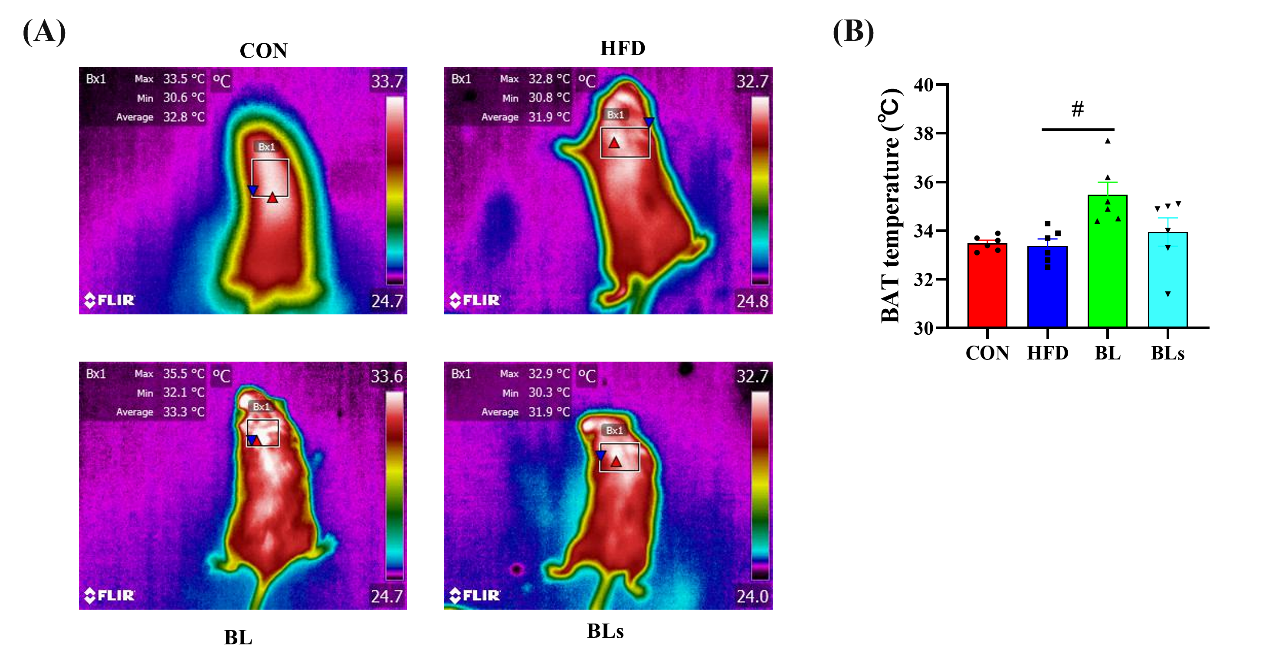


**Supplementary Fig 3** *B. laterosporus* BL1 treatment increased thermogenesis of brown adipose tissue in HFD-fed mice. (A) Representative infra-red thermal image of brown adipose tissue, (B) infrascapular BAT temperature measured by infra-red camera. Data are presented as mean ± SEM (n = 6) and analyzed using one-way ANOVA. Significant differences between HFD and BL are indicated by ^#^p < 0.05.

**Supplementary Table 3** Effects of *B. Laterosporus* BL1 on clean reads and sequence length of microbiota

| #Sample_name | Clean_Reads (#) | Base (nt) | AvgLen (nt) | Min_length | Max_length |
| --- | --- | --- | --- | --- | --- |
| CON_1 | 54944 | 22979635 | 418 | 260 | 444 |
| CON_2 | 53460 | 22104984 | 413 | 258 | 431 |
| CON_3 | 59361 | 24693287 | 416 | 245 | 517 |
| CON_4 | 54419 | 22524277 | 414 | 258 | 452 |
| CON_5 | 56510 | 23575792 | 417 | 258 | 503 |
| CON_6 | 55092 | 22978724 | 417 | 258 | 431 |
| HFD_1 | 60758 | 25640671 | 422 | 236 | 511 |
| HFD_2 | 62561 | 26420614 | 422 | 232 | 431 |
| HFD_3 | 60504 | 25523123 | 422 | 233 | 492 |
| HFD_4 | 60103 | 25473817 | 424 | 258 | 443 |
| HFD_5 | 58265 | 24622943 | 423 | 258 | 456 |
| HFD_6 | 62319 | 26135701 | 419 | 245 | 431 |
| BL_1 | 63321 | 26782837 | 423 | 235 | 444 |
| BL_2 | 58089 | 24377155 | 420 | 216 | 436 |
| BL_3 | 70152 | 29430591 | 420 | 258 | 501 |
| BL_4 | 60003 | 25256339 | 421 | 258 | 481 |
| BL_5 | 68395 | 28757925 | 420 | 258 | 490 |
| BL_6 | 65550 | 27661120 | 422 | 258 | 511 |
| BLs_1 | 53064 | 22100054 | 416 | 219 | 431 |
| BLs_2 | 64113 | 26730672 | 417 | 258 | 508 |
| BLs_3 | 66663 | 27801406 | 417 | 214 | 499 |
| BLs_4 | 58561 | 24692319 | 422 | 251 | 431 |
| BLs_5 | 62476 | 26089189 | 418 | 258 | 431 |
| BLs_6 | 61702 | 25723048 | 417 | 222 | 431 |

**Supplementary Fig. 4**


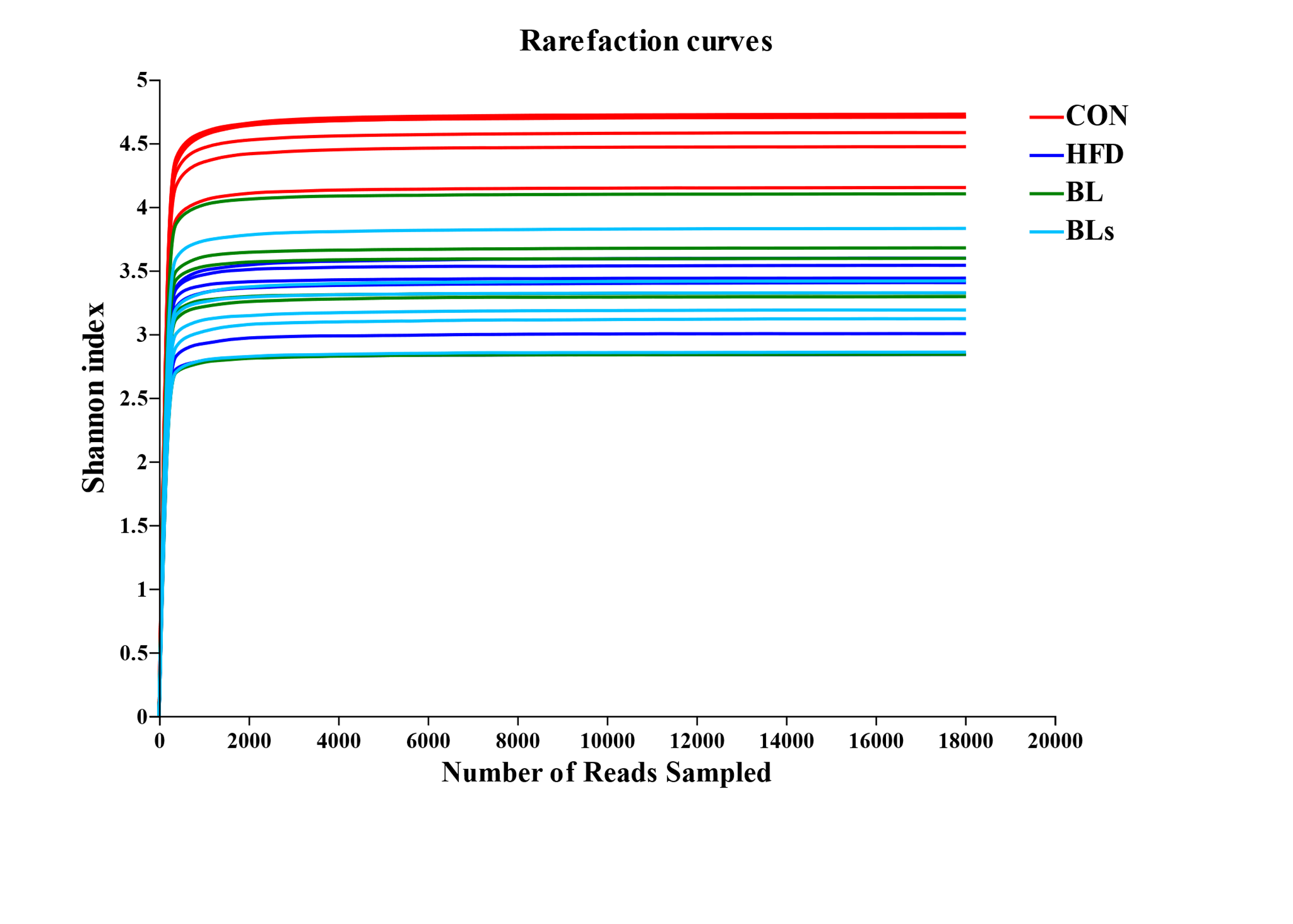


**Supplementary Fig. 4** Rarefaction curves of the 16S rRNA gene reads based on Shannon index. The near saturated rarefaction curve indicates that the vastness of microbial diversity was retrieved from each sample.
